# Quinine and Tannic Acid

**Published:** 1889-08

**Authors:** 


### Quinine and Tannic Acid.

It is said that one and a-half grains of tannin will neutralize the bitterness, without changing the action of ten grains of quinine. The intense bitterness of the drug renders it almost impossible to administer it to children in its natural state.

